# A Feature Selection Algorithm Integrating Maximum Classification Information and Minimum Interaction Feature Dependency Information

**DOI:** 10.1155/2021/3569632

**Published:** 2021-12-28

**Authors:** Li Zhang

**Affiliations:** School of Computer Engineering, Jiangsu University of Technology, Jiangsu, Changzhou 213001, China

## Abstract

Feature selection is the key step in the analysis of high-dimensional small sample data. The core of feature selection is to analyse and quantify the correlation between features and class labels and the redundancy between features. However, most of the existing feature selection algorithms only consider the classification contribution of individual features and ignore the influence of interfeature redundancy and correlation. Therefore, this paper proposes a feature selection algorithm for nonlinear dynamic conditional relevance (NDCRFS) through the study and analysis of the existing feature selection algorithm ideas and method. Firstly, redundancy and relevance between features and between features and class labels are discriminated by mutual information, conditional mutual information, and interactive mutual information. Secondly, the selected features and candidate features are dynamically weighted utilizing information gain factors. Finally, to evaluate the performance of this feature selection algorithm, NDCRFS was validated against 6 other feature selection algorithms on three classifiers, using 12 different data sets, for variability and classification metrics between the different algorithms. The experimental results show that the NDCRFS method can improve the quality of the feature subsets and obtain better classification results.

## 1. Introduction

In the era of big data, the number of dimensions of small sample data has increased dramatically, leading to dimensional disasters. In the preprocessing stage, irrelevant and redundant features need to be processed using data dimension reduction techniques. Because there are a lot of irrelevant and redundant features in high-dimensional data, these features not only lead to higher computational complexity but also reduce the accuracy and efficiency of classification methods. Feature selection [1–5] differs from other data dimensionality reduction techniques (e.g., feature extraction) [6] in that feature selection focuses on analysing the relevance and redundancy in high-dimensional data, removing as many irrelevant and redundant features as possible and retaining the relevant original physical features. This approach not only improves the data quality and classification performance but also reduces the training time of the model and makes it more interpretable [7–9].

Feature selection methods can be classified into three types: filter methods [10, 11], wrapper methods [12], and embedded methods [13]. Due to their high computational efficiency and generality, filter methods are also easily applied to ultra-high-dimensional data sets. In this paper, the filter feature selection method is used. The filter feature selection methods can be classified into rough set [14], statistics-based [15], and information-based [16] according to different metrics. Among these criteria, information-theoretic-based feature selection algorithms are currently the most popular research direction for filter feature selection algorithms. Usually, feature selection algorithms in information theory are further divided into mutual information metrics [17, 18], conditional mutual information metrics [1, 19], interactive mutual information metrics [20–22], and so on. These methods then only determine whether the features are redundant and relevant under a single condition, so the optimal feature subset cannot be obtained. At the same time, the main differences between feature extraction in deep learning and feature selection algorithms based on information-theoretic filtering are described in two ways: (1) from a business perspective, feature selection algorithms can analyse features, whereas feature extraction can only perform pattern mapping and not correlation analysis and research; (2) from an efficiency perspective, feature extraction requires higher computational resources and longer training time, whereas feature selection only needs to be performed in a low-performance server.

In a high-dimensional small sample environment, the dynamic search for redundant and correlated features between features becomes a current problem to be solved in response to the diversity and high dimensionality of the data. This paper proposes a feature selection algorithm for nonlinear dynamic conditional relevance (NDCRFS). The innovations and contributions of this paper are as follows:

1. Firstly, the correlation between independent features and class labels is calculated by mutual information. Secondly, the correlation between the candidate features and the selected features under the class label is calculated using the conditional information. Finally, the correlation and redundancy between features are judged by the interaction information. This method solves the problem of how to measure the relevance and redundancy between selected features and candidate features.
2. The interaction information is normalized by an information gain factor to solve the dynamic balance of interaction information values.
3. Experimental comparison of 12 benchmark data sets in k-nearest neighbour (KNN), decision tree (C4.5), and support vector machine (SVM) classifiers showed that the NDCRFS algorithm outperformed other feature selection algorithms (Mutual Information Maximization (MIM) [23], Interaction Gain-Recursive Feature Elimination (IG-RFE) [24], Interaction Weight Feature Selection (IWFS) [21], Conditional Mutual Information Maximization (CMIM) [25], Dynamic Weighting-based Feature Selection (DWFS) [26], and Conditional Infomax Feature Extraction (CIFE) [23]). The experimental results demonstrate that the proposed NDCRFS algorithm is an effective criterion for classifying feature subsets and can select the feature subsets with good classification performance.

The rest of the paper is organised as follows. In Section 2, related work is presented.  Section 3 discusses mutual information and conditional mutual information. In Section 4, the development of filtered feature selection algorithms is introduced and summarised and also a discussion is given on how to define independent feature relevance and redundancy, new categorical information relevance, and interaction feature dependency relevance and redundancy. In Section 5, the process and details of the implementation of the NDCRFS algorithm are described in detail. In Section 6, the effectiveness of the NDCRFS algorithm is validated by conducting a comprehensive evaluation of 12 data sets in ASU and UCI, while giving a related discussion. In Section 7, the paper is summarised and the shortcomings and future developments of the NDCRFS algorithm are pointed out.

## 2. Mutual Information and Conditional Mutual Information

Let *X*, *Y*, and *Z* be three discrete variables [27], where *X*={*x*_1_, *x*_2_,…, *x*_*L*_}, *Y*={*y*_1_, *y*_2_,…, *y*_*M*_}, *Z*={*z*_1_, *z*_2_,…, *z*_*N*_}. Therefore, the mutual information between *X* and *Y* is defined as follows:

(1) $$\begin{array}{c} I\left(X;Y\right)=\sum_{i=1}^{L}\sum_{j=1}^{M}p\left(x_{i},y_{i}\right)\log_{2}\frac{p\left(x_{i},y_{i}\right)}{p\left(x_{i}\right)p\left(y_{i}\right)}. \end{array}$$

In the above equation, *p*(*x*_*i*_, *y*_*i*_) refers to the joint distribution, and *p*(*x*_*i*_) and *p*(*y*_*j*_) refer to the marginal distribution.

Also, the conditional mutual information of *X* , *Y*, and *Z* is defined as follows:

(2) $$\begin{array}{c} I\left(X;Y|Z\right)=\sum_{t=1}^{N}p\left(z_{t}\right)\sum_{i=1}^{L}\sum_{j=1}^{M}p\left(x_{i},y_{i}|z_{t}\right)\times \;\log_{2}\frac{p\left(x_{i},y_{i}|z_{t}\right)}{p\left(x_{i}|z_{t}\right)p\left(y_{i}|z_{t}\right)}. \end{array}$$

## 3. Related Work

A large number of feature selection algorithms have been proposed for filters, which mainly use forward search to find the optimal subset of features by evaluating the relevance between features and class labels and the redundancy between features using their respective evaluation criteria. Let *F* be the original set of features and let *S* be the best feature subset *S* ⊂ *F*, *J*(·) represents the assessment criteria, *f*_*k*_ indicates candidate features, and *f*_select_ indicates a selected feature, *f*_*k*_ ∈ *F*, *f*_*k*_ ∉ *S*, *f*_select_ ∈ *S*.

Lewis et al. proposed the MIM algorithm, which focuses on selecting the *k* most relevant features from *F* using the relevance of the features to the class labels. In the MIM algorithm, it is evaluated by the following criteria:

(3) $$\begin{array}{c} J_{\text{MIM}}\left(f_{k}\right)=I\left(f_{k};C\right). \end{array}$$

Therefore, Lin et al. studied the limitations of the MIM algorithm and proposed CIFE algorithm, in which it is evaluated with the following criteria:

(4) $$\begin{array}{c} J_{\text{CIFE}}\left(f_{k}\right)=I\left(C;f_{k}\right)-\sum_{f_{\text{select}}\in S}I\left(C;f_{\text{select}};f_{k}\right),\\ =I\left(C;f_{k}\right)-\sum_{f_{\text{select}}\in S}\left\{I\left(f_{k};f_{\text{select}}\right)-I\left(f_{k};f_{\text{select}}|C\right)\right\}. \end{array}$$

In *J*_CIFE_, in addition to measuring redundancy *I*(*f*_*k*_; *f*_*i*_) between features, it is proposed to measure redundancy within class labels *I*(*f*_*k*_; *f*_*i*_*|C*) .

Yang et al. [28] proposed the Joint Mutual Information (JMI) algorithm, which is evaluated with the following criteria:

(5) $$\begin{array}{c} J_{\text{JMI}}\left(f_{k}\right)=I\left(C;f_{k}\right)-\frac{1}{\left|S\right|}\sum_{f_{\text{select}}\in S}I\left(C;f_{\text{select}};f_{k}\right),\\ =I\left(C;f_{k}\right)-\frac{1}{\left|S\right|}\sum_{f_{\text{select}}\in S}\left\{I\left(f_{k};f_{\text{select}}\right)-I\left(f_{k};f_{\text{select}}|C\right)\right\}, \end{array}$$

where *J*_JIM_(*f*_*k*_) has only one additional weighting factor 1/|*S*| over *J*_CIFE_ and |*S*| represents the optimal number of feature subsets.

Fleuret et al. proposed CMIM algorithm according to the maximum-minimum criterion, which is evaluated as follows:

(6) $$\begin{array}{c} J_{\text{CMIM}}\left(f_{k}\right)=I\left(f_{k};C\right)-\max_{f_{\text{select}}\in S}\left(I\left(f_{k};f_{\text{select}}\right)-I\left(f_{k};f_{\text{select}}|C\right)\right). \end{array}$$

The difference between *J*_CMIM_(*f*_*k*_) and *J*_CIFE_(*f*_*k*_) is that *J*_CMIM_(*f*_*k*_) uses a nonlinear cumulative summation standard, while *J*_CIFE_(*f*_*k*_) uses a linear cumulative summation standard.

Sun et al. considered nonlinear criteria with low computational cost and therefore proposed DWFS, in which the DWFS algorithm is evaluated as follows:

(7) $$\begin{array}{c} W_{\text{DWFS}}\left(f_{k}\right)=W_{\text{DWFS}}\left(f_{k}\right)\times \left(2\times \frac{I\left(f_{k};C|f_{\text{select}}\right)-I\left(f_{k};C\right)}{H\left(f_{k}\right)+H\left(C\right)}+1\right), \end{array}$$

where, in the *W*_DWFS_(*f*_*k*_) standard, *I*(*f*_*k*_; *C|f*_select_) > *I*(*f*_*k*_; *C*) means relevant and *I*(*f*_*k*_; *C|f*_select_) < *I*(*f*_*k*_; *C*) means redundant.

Hu et al. [29] proposed the Dynamic Relevance and Joint Mutual Information Maximization (DRJMIM) algorithm based on the DWFS algorithm and the JMIM algorithm, which mainly addresses the definition of feature relevance, that is, how to distinguish between the relevance of candidate features and the relevance of selected features. The evaluation criteria of this algorithm are as follows:

(8) $$\begin{array}{c} J_{\text{DRJMIM}}\left(f_{k}\right)=\min_{f_{\text{select}}\in S}\left(I\left(f_{k};f_{\text{select}};C\right)\right)\times \left(I\left(f_{k};C\right)+C\_\text{Ratio}\left(f_{k},f_{\text{select}}\right)\times I\left(f_{\text{select}};C\right)\right). \end{array}$$

In the above equation, *C*_Ratio(*f*_*k*_, *f*_select_)=2 × (*I*(*f*_*k*_, *C|f*_select_) − *I*(*f*_*k*_, *C*)/*H*(*f*_*k*_)+*H*(*C*)).

Xiao et al. [30] believed that the use of redundancy between features can further improve the accuracy of the classification algorithm. Based on this, the Dynamic Weights Using Redundancy (DWUR) algorithm was proposed. Evaluation criteria of the algorithm are as follows:

(9) $$\begin{array}{c} W_{\text{DWUR}}\left(f_{k}\right)=W_{\text{DWUR}}\left(f_{k}\right)\times \left(I\left(f_{k};C\right)+C\_\text{Ratio}\left(f_{k},f_{\text{select}}\right)\times I\left(f_{\text{select}};C\right)\right). \end{array}$$

In the above equation, *W*_DWUR_(*f*_*k*_) has one more (1 − *β* × *I*(*f*_*k*_; *f*_select_)) item than *W*_DWFS_(*f*_*k*_).

In summary, the analysis of equations (3) to (9) shows that the existing feature selection algorithms all have some of the following problems: (1) Redundant features and irrelevant features are not completely eliminated. (2) Interdependent features are often removed as redundant features because they are highly correlated with each other. These algorithms ignore judgements about the relevance and redundancy of interdependent features. (3) The dependency relevance and redundancy of interaction features can be judged by conditional mutual information and mutual information differences. Therefore, the study of better feature selection criteria is an urgent problem to be solved.

## 4. Evaluation Basis for Feature Selection

Bennasar et al. [31] argued that a feature *f* is considered useful if it is related to the class label *C*; otherwise, feature *f* is considered useless. This assumption only considers features to be completely independent of each other. In reality, feature *f* and label *C* correlations vary with the addition of different features, and it can be concluded that there are interdependencies between features and that feature *f* and class label *C* correlations and redundancies change dynamically with each other. In this section, the relevance and redundancy of independent and dependent features will be analysed and discussed. Let *f*_*j*_ ∈ *F* − *S*, *f*_*i*_ ∈ *F* − *S*, *f*_*i*_ ≠ *f*_*j*_.

### 4.1. Independent Feature Relevance and Redundancy Analysis

Mutual information *I*(*f*_*i*_; *C*) is often used to assess the correlation between feature *f*_*i*_ and the class label *C*. The stronger the correlation between feature *f*_*i*_ and the class label *C* is, the closer the *I*(*f*_*i*_; *C*) value is to 1; conversely, the weaker the correlation is, the closer the value is to 0. If *I*(*f*_*i*_; *C*) > *I*(*f*_*j*_; *C*), then the correlation between feature *f*_*i*_ and the class label *C* is stronger than the correlation between feature *f*_*j*_ and the class label *C*. If *I*(*f*_*i*_; *C*) < *I*(*f*_*j*_; *C*), then the correlation between feature *f*_*i*_ and the class label *C* is weaker than the correlation between feature *f*_*j*_ and the class label *C*.

The mutual information *I*(*f*_*i*_; *f*_*j*_) is often used to assess the correlation between feature *f*_*i*_ and feature *f*_*j*_. If the correlation between *f*_*i*_ and *f*_*j*_ is high, then the redundancy between features is strong; conversely, the redundancy is weak. When *I*(*f*_*i*_; *f*_*j*_)=0, the features *f*_*i*_ and *f*_*j*_ are independent of each other. When *I*(*f*_*i*_; *f*_*j*_)=1, it means that feature *f*_*i*_ and feature *f*_*j*_ are redundant, and then it means that feature *f*_*i*_ or *f*_*j*_ is deleted.

### 4.2. Relevance Analysis of New Classification Information

If *I*(*f*_*i*_; *C|f*_select_) > 0, it means that the candidate feature *f*_*i*_ can provide more classification information. If *I*(*f*_*i*_; *C|f*_select_)=0, it means that the candidate feature *f*_*i*_ cannot provide any useful classification information and the features *f*_*i*_ and *f*_select_ are independent of each other.

If *I*(*f*_*i*_; *C|f*_select_) > *I*(*f*_*j*_; *C|f*_select_), it means that feature *f*_*i*_ provides more classification information than feature *f*_*j*_.

### 4.3. Relevance and Redundancy of Interaction Feature Dependencies

According to the literature [6, 18, 29], if *I*(*f*_*i*_; *f*_select_*|C*) > *I*(*f*_select_; *C*) relevance of the selected feature *f*_select_ to the class label *C* is becoming stronger after the candidate feature *f*_*i*_ is added, it indicates that the candidate feature *f*_*i*_ can provide more classification information.

If *I*(*f*_*i*_; *f*_select_*|C*) < *I*(*f*_select_*|C*), the correlation between the selected feature *f*_select_ and the class label *C* is weakening after the candidate feature *f*_*i*_ is added, indicating that the candidate feature *f*_*i*_ and the selected feature *f*_select_ are redundant with each other.

## 5. NDCRFS Algorithm Description and Pseudocode Implementation

The feature selection algorithm seeks to search for sets of features that are closely related to class labels. To more accurately measure the relevance of features to class labels, the NDCRFS algorithm measures the relevance and redundancy of features in three ways:

1. *I*(*f*_*k*_; *C*) to measure the relevance of feature *f*_*k*_ to class label *C*
2. *I*(*f*_*k*_; *f*_select_*|C*) to measure the relevance of feature *f*_*k*_ to the selected feature *f*_select_ under class label *C*
3. *I*(*f*_*k*_; *f*_select_*|C*) − *I*(*f*_select_; *C*) measuring the interaction correlation and redundancy between *f*_*k*_ and *f*_select_ under the class label *C*

Therefore, for the evaluation criteria for the NDCRFS algorithm, the specific formula is as follows:

(10) $$\begin{array}{c} J_{\text{NDCRFS}}\left(f_{k}\right)=I\left(f_{k};C\right)-\max_{f_{\text{select}}\in S}CU\left(f_{\text{select}},f_{k}\right)\times \left[I\left(f_{k};f_{\text{select}}|C\right)-I\left(f_{\text{select}};C\right)\right]. \end{array}$$

In the above formula, *CU*(*f*_select_, *f*_*k*_)=2/*H*(*f*_select_*|C*)+*H*(*f*_*k*_*|C*), *CU*(*f*_select_, *f*_*k*_) is used as an information gain factor to normalize *I*(*f*_*k*_; *f*_select_*C*) − *I*(*f*_select_; *C*).*f*_*k*_ indicates candidate features and *f*_select_ indicates a selected feature, *f*_*k*_ ∈ *F*, *f*_*k*_ ∉ *S*, *f*_select_ ∈ *S*.

From equation (10), in the NDCRFS algorithm, it firstly selects the minimum redundant features from *J*_NDCRFS_(*f*_*k*_) based on the correlation analysis between the selected features *f*_select_ and the candidate features *f*_*k*_; secondly, it selects the most relevant features to the optimal feature subset *S* by iteration, and its pseudocode is as follows.

From Algorithm 1, line 1 initializes set *S* and counters *k*. In lines 2 to 7, the mutual information of each feature in the set *F* is calculated. In lines 8 to 10, at the same time, the selected optimal feature *f*_*k*_ is removed from set *F*, and feature *f*_*k*_ is added to set *S*. At this time, the candidate feature *f*_*k*_ becomes the selected feature *f*_select_. In lines 11 to 18, the values of *I*(*f*_*k*_; *C|f*_select_), *I*(*f*_*k*_; *f*_select_*|C*), and *I*(*f*_select_; *C*) are calculated.

The NDCRFS algorithm consists of 2 “for” loops and 1 “while” loop. Therefore, the time complexity of the NDCRFS algorithm is *O*(*Tmn*) (*T* represents the number of selected features, *n* represents the number of all features, and *m* represents the number of all samples, where *T* ≪ *n*). The complexity of the NDCRFS algorithm is higher than that of the MIM algorithm, IWFS algorithm, CMIM algorithm, DWFS algorithm, and CIFE algorithm, but the NDCRFS algorithm is lower than the IG-RFE algorithm, mainly because the NDCRFS algorithm also needs to calculate *CU*(*f*_select_, *f*_*k*_), *I*(*f*_*k*_; *f*_select_*|C*) − *I*(*f*_select_; *C*), *I*(*f*_*k*_; *C|f*_select_).

## 6. Experiments and Results

### 6.1. Introduction to the Data Set

In order to verify the effectiveness of the NDCRFS algorithm, a total of 12 data sets were used in the experiments. The experimental data sets were selected from the internationally renowned UCI [3] and ASU [14] general data sets, which are described in detail in Table 1. From Table 1, we know that the sample range is from 60 to 7494, the feature range is from 16 to 19 993, and the classification label range is from 2 to 20. The experimental data sets involve biomedical (Lymphography, Dermatology, Lung Cardiotocography, Lymphoma, Nci9, SMK-CAN-187, and Carcinom), face image data (COIL20 and Pixraw10P), and text data (PCMAC and Pendigits).

### 6.2. Experimental Environment Setup

NDCRFS was compared with six feature selection algorithms, MIM, IG-RFE, IWFS, CMIM, DWFS, and CIFE, to verify its effectiveness. The experiments were conducted using KNN, SVM, and C4.5, respectively, on the same feature subsets. The number of feature subsets was set as (*K*); for example, *K* = 10 for Lymphography and Pendigits and *K*=30 for the rest of the settings. The experimental environment for this paper was an Intel-i7 processor with 8 GB RAM, and the simulation software was Python 2.7. A 5-fold cross-validation method was used in the experiments to obtain the average classification accuracy of the current classifier for that feature selection algorithm's average classification accuracy. In the experiment, the incomplete samples are deleted, and, at the same time, according to Kuarga [32], the class attribute dependence maximization method is used to discretize continuous data.

### 6.3. Discussion and Analysis of Experimental Results

#### 6.3.1. Comparison of Algorithm Variability

This paper proposes a method to measure the difference between two selected feature subsets using the Jaccard method. Among them, *S*_1_ ⊂ *F*, *S*_2_ ⊂ *F*, *S*_1_ ≠ *S*_2_.*S*_1_ represents the feature subset selected by the NDCRFS algorithm, and *S*_2_ represents the feature subset selected by other feature selection algorithms. The specific formula (11) is as follows:

(11) $$\begin{array}{c} \text{Jaccard}\left(S_{1},S_{2}\right)=\frac{\left|S_{1}\cap S_{2}\right|}{\left|S_{1}\cup S_{2}\right|}. \end{array}$$

As can be seen in Table 2, the mean values of the difference between NDCRFS and MIM, NDCRFS and IG-RFE, NDCRFS and IWFS, NDCRFS and CMIM, NDCRFS and DWFS, and NDCRFS and CIFE are 0.355, 0.389, 0.261, 0.222, 0.286, and 0.166, respectively, indicating that the difference between features is not considered. When sorting the relationship, the NDCRFS algorithm is significantly different from the other feature selection algorithms.

### 6.4. Comparison of Classification Accuracy

Tables 3to 5 show the average classification accuracy on the 12 data sets using KNN, C4.5, and SVM. Bold represents the highest accuracy value in the feature selection algorithm for that data set. Tables 3–5 show that the NDCRFS algorithm had the highest average classification accuracy of 88.734%, 81.574%, and 79.213%, respectively. “Wins/Ties/Losses” describes the number of wins/ties/losses between NDCRFS and MIM, IG-RFE, IWFS, CMIM, DWFS, and CIFE.

**Figure 1:**
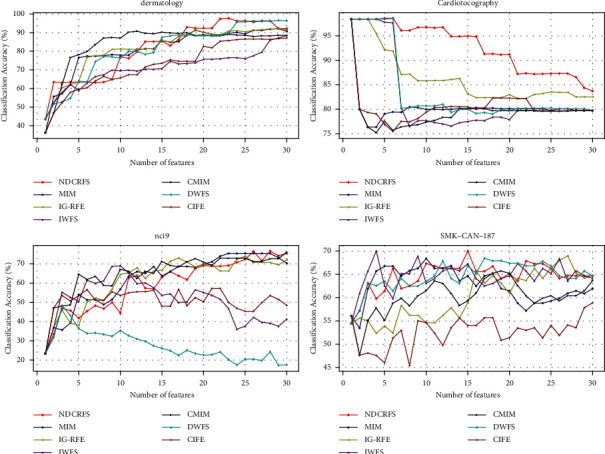
Comparison of accuracy in KNN classifier.

From Table 3, it is clear that the NDCRFS algorithm outperforms the MIM, IG-RFE, IWFS, CMIM, DWFS, and CIFE algorithms in most data sets by 12, 12, 12, 12, 12, and 12, respectively. In Figure 1(a), the classification accuracy of the NDCRFS algorithm is the highest compared to the six classification algorithms (97.769%, the required number of features is 23), which is 5.605%, 5.605%, 9.257%, 6.979%, 1.089%, and 10.63% higher, respectively. In Figure 1(b), the classification accuracy of the NDCRFS algorithm is the highest compared to the six classification algorithms (98.589%, the number of required features is 5), which is 0.188%, 0.188%, 0.188%, 0.188%, 0.0%, and 0.188% higher, respectively. In Figure 1(c), the classification accuracy of the NDCRFS algorithm is the highest compared to the six classification algorithms (76.69%, the required number of features is 28), which is 1.25%, 2.678%, 7.666%, 0.571%, 28.261%, and 19.44% higher, respectively. In Figure 1(d), the classification accuracy of the NDCRFS algorithm is the highest compared to the six classification algorithms (70.014%, the number of required features is 15), which is 1.621%, 1.01%, 0.014%, 4.267%, 1.593%, and 11.138% higher, respectively.

**Figure 2:**
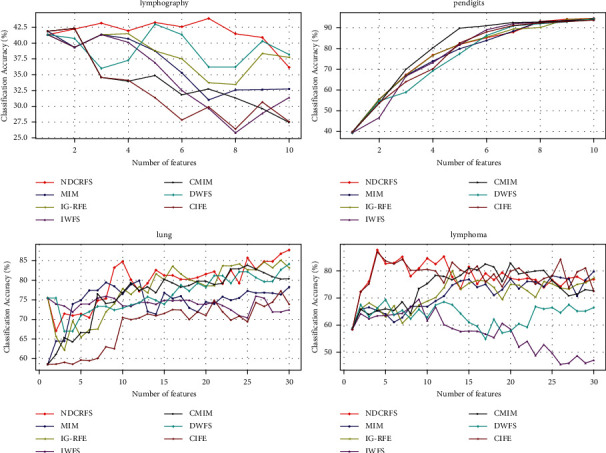
Comparison of accuracy in C4.5 classifier.

From Table 4, the NDCRFS algorithm is superior to the MIM, IG-RFE, IWFS, CMIM, DWFS, and CIFE algorithms in the majority of data sets, with 11, 11, 11, 10, 10, and 11, respectively. In Figure 2(a), the classification accuracy of the NDCRFS algorithm is the highest compared to the six classification algorithms (43.935%, the required number of features is 7), which is 2.042%, 2.462%, 2.588%, 1.613%, 0.933%, and 1.613% higher, respectively. In Figure 2(b), the classification accuracy of the NDCRFS algorithm is the highest compared to the six classification algorithms (94.569%, the number of required features is 10), which is 0.226%, 0.373%, 0.787%, 0.801%, 0.347%, and 0.894% higher, respectively. In Figure 2(c), the classification accuracy of the NDCRFS algorithm is the highest compared to the six classification algorithms (87.774%, the required number of features is 30), which is 7.856%, 2.661%, 11.81%, 3.932%, 3.617%, and 10.538% higher, respectively. In Figure 2(d), the classification accuracy of the NDCRFS algorithm is the highest compared to the six classification algorithms (87.75%, the required number of features is 4), which is 8.0%, 7.75%, 18.222%, 4.944%, 18.333%, and 0.833% higher, respectively.

**Figure 3:**
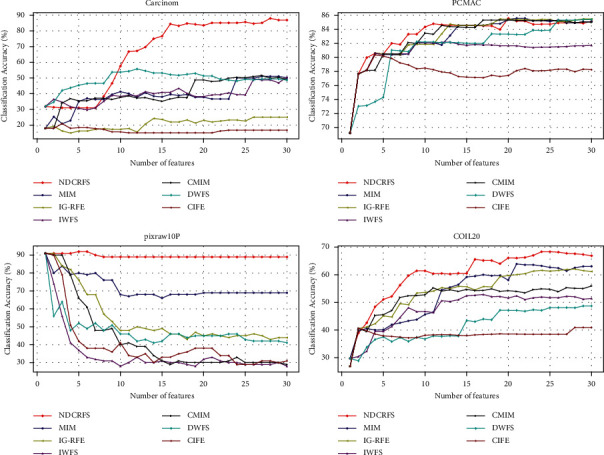
Comparison of accuracy in SVM classifier.

From Table 5, the NDCRFS algorithm is superior to the MIM, IG-RFE, IWFS, CMIM, DWFS, and CIFE algorithms in the majority of data sets, with 10, 12, 12, 11, 10, and 11, respectively. In Figure 3(a), the classification accuracy of the NDCRFS algorithm is the highest compared to the six classification algorithms (87.964%, the number of required features is 28), which is 36.966%, 62.936%, 37.517%, 36.419%, 32.191%, and 67.049% higher, respectively. In Figure 3(b), the classification accuracy of the NDCRFS algorithm is the highest compared to the six classification algorithms (85.589% with 20 required features), which is 0.001%, 0.102%, 3.394%, 0.255%, 0.206%, and 5.194% higher, respectively. In Figure 3(c), the classification accuracy of the NDCRFS algorithm is the highest compared to the six classification algorithms (92%, the number of required features is 5), which is 1%, 1%, 1%, 1%, 1%, and 1% higher, respectively. In Figure 3(d), the classification accuracy of the NDCRFS algorithm is the highest compared to the six classification algorithms (68.352%, the number of features required is 24), which is 4.466%, 6.285%, 15.528%, 12.419%, 19.714%, and 27.447% higher, respectively.

### 6.5. Runtime Analysis of the Algorithm

Calculating the running time of feature selection algorithms is also one of the criteria to measure the importance of feature selection algorithms. Now, the running times of the NDCRFS algorithm, the MIM algorithm, the IG-RFE algorithm, the IWFS algorithm, the CMIM algorithm, the DWFS algorithm, and the CIFE algorithm are compared. In Table 6, these feature selection algorithms are the final runtimes derived from the feature ranking of all features of the 12 data sets. The NDCRFS algorithm's runtimes are well within acceptable limits.

The results of the 5-fold cross-validation experiments on the ASU and UCI data sets show that the proposed NDCRFS algorithm is able to select a subset of features with better classification performance, which can further improve the discrimination ability of the data set under data dimensionality compression.

## 7. Conclusion

Feature selection is an important tool for the data preprocessing phase in high-level small sample data. The main objective of feature selection is to select the optimal subset of features and should have a high classification accuracy. Therefore, in this paper, a nonlinear dynamic conditional correlation feature selection algorithm is proposed. The algorithm first uses mutual information, conditional mutual information, and interactive mutual information to determine and identify the relevance and redundancy of independent features and dependent features. Secondly, the “max-min” principle is used to eliminate redundant and irrelevant features from the original feature set iteratively. Finally, the effectiveness of this algorithm is verified through experiments, which demonstrate that the NDCRFS algorithm significantly outperforms feature selection algorithms MIM, IG-RFE, IWFS, CMIM, DWFS, and CIFE in most of the data sets.

However, the NDCRFS algorithm also has an unsatisfactory selection of feature subsets on some data sets. In the future, it will be necessary to optimize the NDCRFS, while verifying the proposed method in research fields.

## Figures and Tables

**Algorithm 1:**
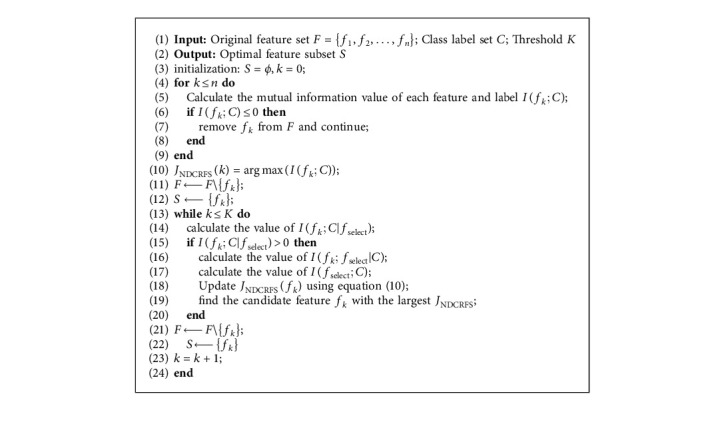
NDCRFS algorithm.

**Table 1:** Experimental data set description.

No.	Data set	Samples	Features	Categories	Data sources
1	Lymphography	148	18	8	UCI
2	Dermatology	358	34	6	UCI
3	Cardiotocography	2126	41	3	UCI
4	Pendigits	7494	16	10	UCI
5	Lung	203	3312	5	ASU
6	Carcinom	174	9182	11	ASU
7	Nci9	60	9712	9	ASU
8	PCMAC	1943	3289	2	ASU
9	Pixraw10P	100	10,000	10	ASU
10	SMK-CAN-187	187	19,993	2	ASU
11	Lymphoma	96	4026	9	ASU
12	COIL20	1440	1024	20	ASU

**Table 2:** The difference between NDCRFS and the comparison algorithms.

No.	MIM	IG-RFE	IWFS	CMIM	DWFS	CIFE
1	0.667	0.818	0.333	0.333	0.429	0.176
2	0.935	0.935	0.765	0.765	0.818	0.765
3	0.538	0.579	0.5	0.463	0.5	0.5
4	0.818	0.818	0.333	0.333	0.25	0.25
5	0.017	0.017	0.017	0.0	0.132	0.0
6	0.0	0.017	0.017	0.0	0.034	0.091
7	0.579	0.622	0.053	0.224	0.017	0.034
8	0.429	0.5	0.224	0.395	0.25	0.091
9	0.034	0.017	0.017	0.017	0.091	0.017
10	0.091	0.017	0.818	0.0	0.765	0.0
11	0.132	0.132	0.034	0.132	0.071	0.071
12	0.017	0.2	0.017	0.0	0.071	0.0
Average	0.355	0.389	0.261	0.222	0.286	0.166

**Table 3:** Average classification accuracy (%) of KNN classifier.

Data set	NDCRFS	MIM	IG-RFE	IWFS	CMIM	DWFS	CIFE
Lymphography	**38.3**	34.78	35.59	35.59	34.88	35.28	34.78
Dermatology	**97.769**	92.164	92.164	88.512	90.79	96.68	87.139
Cardiotocography	**98.589**	98.401	98.401	98.401	98.401	98.589	98.401
Pendigits	**97.919**	97.145	97.145	97.238	97.505	98.159	97.625
Lung	**88.636**	88.064	83.712	76.391	81.678	87.681	74.922
Carcinom	**85.48**	68.037	32.255	60.035	65.84	67.026	31.952
Nci9	**76.69**	75.44	74.012	69.024	76.119	48.429	57.25
PCMAC	**87.648**	85.538	86.155	82.348	84.765	85.743	78.952
Pixraw10P	**93.0**	88.0	91.0	88.0	92.0	88.0	92.0
SMK-CAN-187	**70.014**	68.393	69.004	70.0	65.747	68.421	58.876
Lymphoma	**95.667**	84.722	84.75	69.806	90.083	72.056	82.833
COIL20	**84.662**	80.733	79.743	71.667	77.114	72.024	60.652
Average accuracy rate	**88.734**	84.24	76.994	75.584	83.64	76.507	71.28
Wins/Ties/Losses		12/0/0	12/0/0	12/0/0	12/0/0	12/0/0	12/0/0

The “Average” column gives the average accuracy value of the feature selection algorithm over all datasets. Bold represents the highest average classification prediction under this dataset.

**Table 4:** Average classification accuracy (%) of C4.5 classifier.

Data set	NDCRFS	MIM	IG-RFE	IWFS	CMIM	DWFS	CIFE
Lymphography	**43.935**	41.893	41.473	41.347	42.322	43.002	42.322
Dermatology	**95.021**	94.434	94.149	94.187	95.021	93.337	94.727
Cardiotocography	**98.401**	98.401	98.401	98.401	98.401	98.401	98.401
Pendigits	**94.569**	94.343	94.196	93.782	93.768	94.222	93.675
Lung	**87.774**	79.918	85.113	75.964	83.842	84.157	77.236
Carcinom	**70.604**	54.586	25.79	48.292	56.822	53.999	24.3
Nci9	69.929	61.012	65.095	60.667	**71.083**	57.929	60.226
PCMAC	**87.906**	86.464	86.515	82.502	85.897	86.669	80.805
Pixraw10P	**99.0**	97.0	96.0	92.0	95.0	92.0	95.0
SMK-CAN-187	64.125	62.006	61.494	63.656	62.077	**65.747**	57.852
Lymphoma	**87.75**	79.75	80.0	69.528	82.806	69.417	86.917
COIL20	**79.876**	67.614	72.762	63.186	62.895	70.629	58.295
Average accuracy rate	**81.574**	76.452	75.082	73.626	77.495	75.792	72.48
Wins/Ties/Losses		11/1/0	11/1/0	11/1/0	10/1/1	10/1/1	11/1/0

**Table 5:** Average classification accuracy (%) of SVM classifier.

Data set	NDCRFS	MIM	IG-RFE	IWFS	CMIM	DWFS	CIFE
Lymphography	**45.147**	42.499	43.329	41.45	42.825	43.329	42.825
Dermatology	**98.317**	93.777	93.824	93.283	94.079	97.761	93.53
Cardiotocography	**98.448**	98.401	98.401	98.401	98.401	98.401	98.401
Pendigits	**63.331**	63.331	63.331	55.35	59.741	56.979	57.219
Lung	84.788	77.89	78.391	77.891	**86.203**	85.311	77.402
Carcinom	**87.964**	50.998	25.028	50.447	51.545	55.773	20.915
Nci9	76.512	**78.119**	76.69	62.595	74.429	57.929	58.821
PCMAC	**85.589**	85.588	85.486	82.194	85.333	85.382	80.394
Pixraw10P	**92.0**	91.0	91.0	91.0	91.0	91.0	91.0
SMK-CAN-187	70.982	70.569	62.532	**71.593**	65.32	71.053	57.255
Lymphoma	85.5	81.278	79.611	67.056	81.972	72.194	**86.194**
COIL20	**68.352**	63.886	62.067	52.824	55.933	48.638	40.905
Average accuracy rate	**79.213**	73.363	71.641	70.226	73.898	71.979	65.333
Wins/Ties/Losses		10/1/1	12/0/0	12/0/0	11/0/1	10/0/2	11/0/1

The “Average” column gives the average accuracy value of the feature selection algorithm over all datasets. Bold represents the highest average classification prediction under this dataset.

**Table 6:** The runtimes of different feature selection algorithms.

Date set	Runtime (s)						
	NDCRFS	MIM	IG-RFE	IWFS	CMIM	DWFS	CIFE
Lymphography	0.141	0.089	0.171	0.078	0.062	0.078	0.09
Dermatology	1.373	0.712	1.576	0.811	0.671	0.843	0.824
Cardiotocography	9.952	5.976	12.215	6.303	5.523	6.38	5.599
Pendigits	5.725	4.177	6.568	3.588	3.198	3.807	3.878
Lung	216.292	155.033	322.127	134.73	127.425	166.766	131.861
Carcinom	629.731	577.148	744.337	351.026	315.636	407.857	502.515
Nci9	149.744	130.371	167.166	100.876	81.922	104.424	133.998
PCMAC	1206.53	1130.49	1689.445	878.968	615.348	836.969	1133.675
Pixraw10P	335.022	242.977	415.235	216.42	188.65	171.897	259.263
SMK-CAN-187	1649.124	731.813	1905.724	812.859	727.913	995.003	749.035
Lymphoma	102.755	45.368	113.09	96.2	43.591	94.084	248.495
COIL20	414.124	307.934	570.717	290.075	273.888	264.382	248.495
Average	393.376	277.674	495.698	240.995	198.652	254.374	284.811

## Data Availability

The experimental data set selects the world-famous UCI universal data set (https://archive.ics.uci.edu/ml/datasets.html) and the world-famous ASU universal data set http://featureselection.asu.edu/datasets.php).
